# Stage I placental site trophoblastic tumor with complete response after pembrolizumab

**DOI:** 10.1016/j.gore.2026.102023

**Published:** 2026-01-05

**Authors:** Pierre Descargues, Lua R. Eiriksson, Pascal Rousset, Adrien Msika, Benoit You, Alexis Trecourt, Jerome Massardier, Touria Hajri, Bruno Borghese, François Golfier, Pierre-Adrien Bolze, Jérôme Alexandre

**Affiliations:** a Centre Français de Référence des Maladies Trophoblastiques, Hospices Civils de Lyon, Hôpital Lyon Sud, 69495 Pierre Bénite, France; b Service de Chirurgie Gynécologique et Oncologique, Obstétrique, Hospices Civils de Lyon, Hôpital Lyon Sud, 69495 Pierre Bénite, France; c Division of Gynecologic Oncology, Juravinski Hospital and Cancer Centre, Hamilton Health Sciences, Hamilton, Canada; d Department of Obstetrics and Gynecology, McMaster University, Hamilton, Canada; e Department of Radiology, Hospices Civils de Lyon, Lyon Sud University Hospital, Lyon 1 Claude Bernard University, EMR 3738, Pierre Bénite, France; f Université de Lyon, Université Claude Bernard Lyon 1, Faculté de Médecine Lyon-Sud, CICLY, Lyon, France; g Medical Oncology, Institut de Cancérologie des Hospices Civils de Lyon, Centre d’Investigation de Thérapeutiques en Oncologie et Hématologie de Lyon, Centre Hospitalier Lyon-Sud, Hospices Civils de Lyon, Lyon, France; h Hospices Civils de Lyon, Centre Hospitalier Lyon-Sud, Service de Pathologie Multi-Site, Pierre Bénite, France; i Université Claude Bernard Lyon-1, Faculté de Médecine Lyon Sud, Centre pour l’innovation en cancérologie de Lyon (CICLY), UR 3738, Lyon, France; j Department of Obstetrics and Gynecology, University Hospital Femme Mere Enfant, University of Lyon 1, 51, Boulevard Pinel, 69500 Bron, France; k Centre de Recherche des Cordeliers, “Equipe labélisée Ligue Contre le Cancer”, Sorbonne Université, Université de Paris, INSERM, France; l Université de Paris, Paris, France; m Institut du Cancer Paris CARPEM, AP-HP, APHP.Centre, Department of Gynecological Surgery, Hopital Cochin, Paris, France; n Centre de Référence des Maladies Trophoblastiques, Lyon, France; o Service de Chirurgie Gynécologique et Oncologique, Obstétrique, Centre Hospitalier Lyon Sud, Hospices Civils de Lyon, Pierre-Bénite, France; p Institut du Cancer Paris CARPEM, AP-HP, APHP.Centre, Department of Medical Oncology, Hopital Cochin, Paris, France

## Abstract

• Placental site trophoblastic tumors (PSTT) express PD-L1, making them ideal targets for PD-1 and PD-L1 inhibitors.
• For this PSTT patient seeking fertility-sparing treatment, PD-1 inhibition demonstrated complete and sustained response.
• Despite anti PD-1 toxicity, there is potential for significant benefit when no alternative fertility-sparing options exists.

## 1 Introduction

Placental site trophoblastic tumors (PSTT) are rare entities accounting for 1 to 2 % of gestational trophoblastic neoplasias (GTN) (Gadducci et al., 2019). These tumors are typically chemo-resistant such that the accepted standard-of-care treatment for stage I disease is total hysterectomy, even for patients of child-bearing age. Non-standard-of-care fertility-sparing treatment options for patients with PSTT have been rarely described, namely hysteroscopic resection.

Programmed cell death receptor 1 (PD-1), present on T cells, B cells, macrophages, dendritic cells, monocytes, and natural killer cells binds to programmed cell death ligand 1 (PD-L1) and inhibits cytotoxic T effector cell function (Ishida et al., 1992, Keir et al., 2008, Han et al., 2020). PD-L1 is highly expressed in GTN, and particularly in PSTT (Han et al., 2020, Bolze et al., 2017, Veras et al., 2017, Lu et al., 2019). Monoclonal antibodies inhibiting PD-1 (*e.g.,* pembrolizumab) would be expected to demonstrate activity in these tumor types. Only one case of PSTT treated exclusively with anti-PD-1 therapy is reported in the literature; a young patient with PSTT who declined both hysterectomy and standard chemotherapy was successfully treated with pembrolizumab and achieved a subsequent term delivery (Polnaszek et al., 2021). This case report by Polnaszek *et al.,* published in 2021, potentially offers a new treatment option for young patients with a diagnosis of PSTT who wish to maintain fertility, although evidence is limited to this isolated case report and reported duration of follow-up.

## 2 Case

We report the case of a 26-year old woman diagnosed with a PSTT following delivery of her first pregnancy at term. The infant died at 4 months of age due to a genetic kidney disease. Seven months after delivery the patient developed metrorrhagia with a positive serum hCG level of 50 IU/L. She received two intra-muscular injections of methotrexate (MTX) 1 mg/kg for a suspected ectopic pregnancy. Subsequent surveillance demonstrated a progressive rise in hCG.

**Fig. 1:**
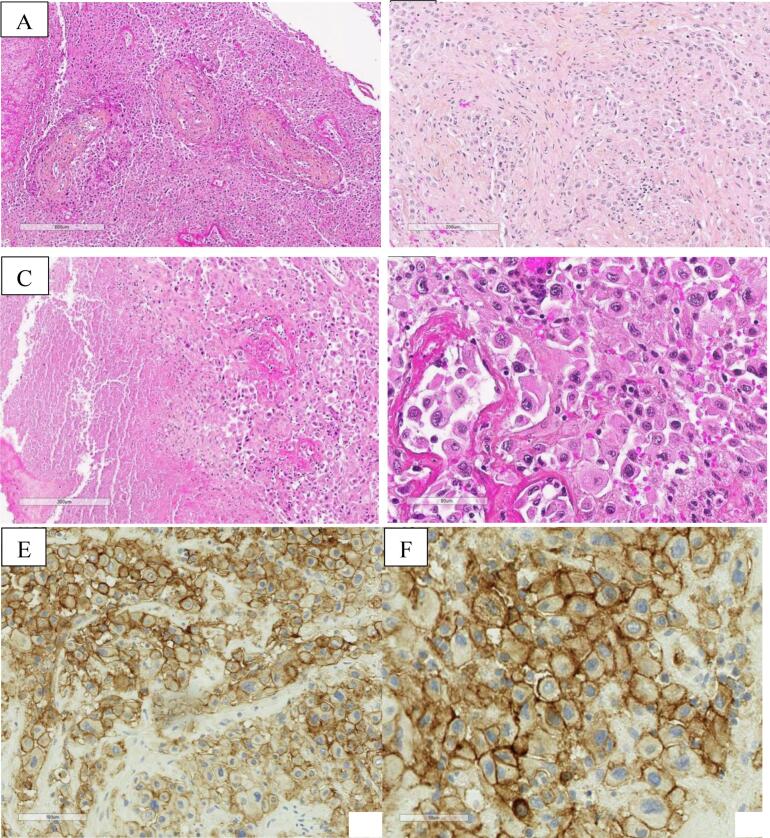
Placental Site Trophoblastic Tumor − Histopathological Findings. A – HES x 50 magnification: Poorly circumscribed proliferation, organized as solid areas of epithelioid cells; presence of characteristic vascular invasion, with replacement of the vascular wall and fibrinoid necrosis; B – HES x 210 magnification: The tumor infiltrates the myometrium, dissecting smooth muscle bundles without tumor stromal reaction; C – HES x 85 magnification: Presence of tumor necrosis; D – HES x 300 magnification: Tumor cells are epithelioid with abundant eosinophilic cytoplasm and atypical, hyperchromatic, pleomorphic nuclei; no syncytiotrophoblastic component is observed; E & F – IHC x 200 and x400 magnification: Intense and diffuse membrane expression of PD-L1 by 80 % of tumor cells. HES: Hematoxylin-eosin-saffron staining; IHC: Immunohistochemistry.

The patient underwent a suction-curettage and laparoscopy. Serum hCG at this time was 79 IU/L. Pathological analysis of the endometrial curettings revealed typical morphological features of PSTT, showing aggregates of large tumor cells with abundant eosinophilic cytoplasm and atypical nuclei infiltrating the myometrium by separating the smooth muscle cells (Fig. 1). Immunohistochemistry (IHC) demonstrated positive diffuse and intense staining for AE1/AE3, GATA3, PD-L1, and hPL without expression of p63, and with a low Ki-67 index. The diagnosis was confirmed by two referent pathologists from the French Reference Center for Trophoblastic Disease (FRCTD). Short tandem repeat genotyping confirmed the gestational origin of the tumor.

Imaging included magnetic resonance imaging (MRI) of the pelvis, computed tomography (CT) of the chest, abdomen, and pelvis, and positron emission tomography with computed tomography (PET/CT).

**Fig. 2:**
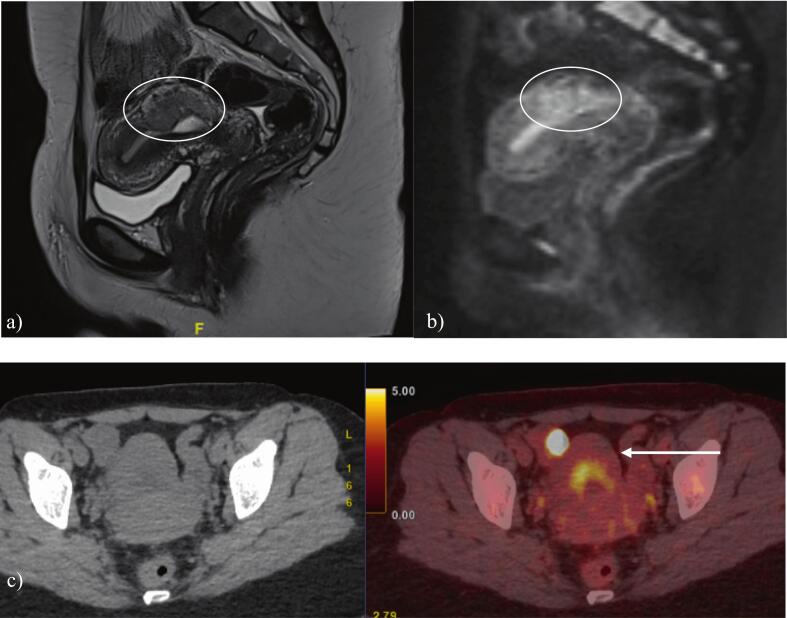
A) pelvic mri with sagittal t2 sequence; b) after gadolinium injection; c) pet/ct confirming 43 mm hypermetabolic mass corresponding to PSTT without distant metastasis.

Pelvic MRI, reviewed by an expert radiologist from the FRCTD, showed a gadolinium-enhancing 43 mm posterior corporeal isthmic myometrial mass. PET/CT confirmed a hypermetabolic uterine mass (SUVmax 5.6) coexisting with a hypermetabolic focus on the right ovary (SUVmax 13.2) corresponding to a corpus luteum cyst (Fig. 2). CT demonstrated no evidence of metastatic disease.

Given the patient’s desire for fertility preservation, the case was discussed at the multi-disciplinary team meeting (MDT) of the FRCTD and surgical resection was offered. The patient underwent operative hysteroscopy, however the surgical resection could not be performed since the tumor was not visible from within the endometrial cavity.

The case was rediscussed at the MDT and two treatment options were proposed: total hysterectomy as the standard-of-care treatment or a trial of fertility-sparing treatment with pembrolizumab after confirmation of stage I disease, based on the case report of Polnaszek *et al.* (Polnaszek et al., 2021) The patient preferred the fertility-sparing option and underwent laparoscopy and bilateral pelvic sentinel lymph node biopsy which revealed no evidence of metastasis. The patient received one intravenous 200 mg dose of pembrolizumab. Serum hCG at that time was 35 IU/L. Three weeks later the patient was diagnosed with bilateral pulmonary emboli and a grade 2 auto-immune myositis which contraindicated on-going treatment. She had no prior history of auto-immune disease.

**Fig. 3:**
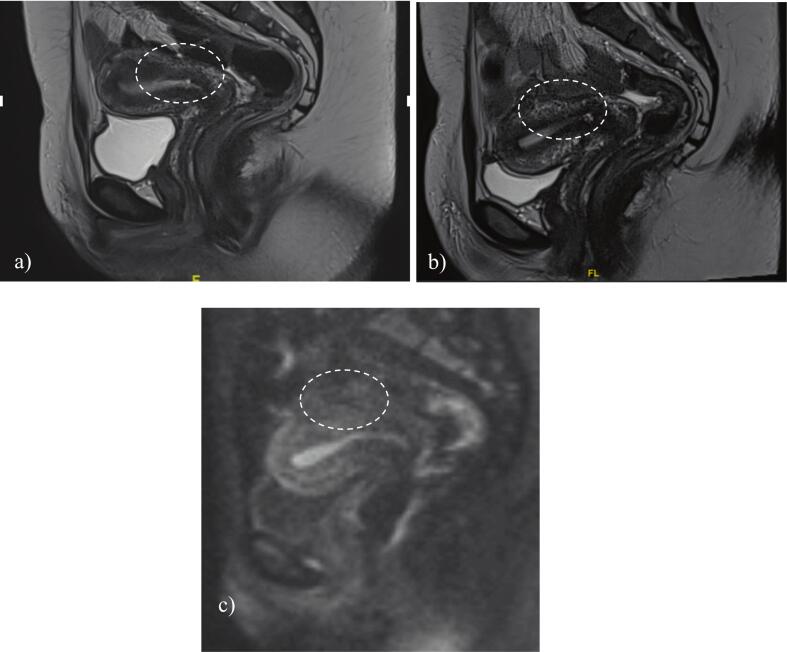
a) pelvic mri two months after treatment with pembrolizumab; b,c) Pelvic MRI 13 months after treatment with pembrolizumab.

Five weeks after receiving pembrolizumab the hCG level normalized. (*N.B.,* Total hCG was measured using an Elecsys hCG immunoassay performed on a Roche cobas e analyzer.) Post-treatment MRI performed two months after receiving pembrolizumab showed a complete response with restoration of normal uterine anatomy, which was confirmed on subsequent imaging (Fig. 3). Repeat PET-CT two months post-treatment demonstrated resolution of the hypermetabolic lesion.

Follow-up included monthly serum hCG level and MRI pelvis every three months. At 34 months following treatment the patient continues to be in remission with no radiologic evidence of disease recurrence on MRI pelvis and CT of the chest, abdomen, and pelvis with a normal hCG (<0.6 IU/L). No pregnancy has been reported to date.

## 3 Discussion

Treatment targeting the PD-1/PD-L1 pathway has demonstrated improvements in response and survival in phase II and phase III clinical trials in oncology including treatment for cervical cancer, lung cancer, and melanoma (Colombo et al., 2021, Mok et al., 2019, Robert et al., 2015, Kwok et al., 2016). Since GTN are known to strongly express PD-L1, there is a strong rationale for treatment with PD-1/PD-L1 inhibitors. The anti-PD-1 antibody pembrolizumab has demonstrated efficacy in the treatment of chemo-resistant GTN (Ghorani et al., 2019). In a literature review by Mangili *et al.,* 66.7 % of patients with chemotherapy-resistant or metastatic GTN treated with pembrolizumab achieved a complete response (Mangili et al., 2022). In the TROPHIMMUN trial, conducted by You *et al.,* the anti-PD-L1 antibody avelumab achieved a response in 53 % of patients with methotrexate-resistant low-risk GTN (You et al., 2020, You et al., 2023).

This is the second case report of immunotherapy in the fertility-sparing treatment of stage I PSTT. The first patient, described by Polnaszek *et al.,* received 3 cycles of pembrolizumab, 200 mg every two weeks, with normalization of the hCG level. The patient became pregnant just before the fourth planned cycle of pembrolizumab and delivered a full-term healthy newborn. Post-partum follow-up included hCG monitoring and CT imaging which confirmed a sustained complete response. In our case, the patient was intended to receive pembrolizumab until normalization of the hCG level followed by five consolidation cycles. With the development of bilateral pulmonary emboli and grade 2 auto-immune myositis after the first cycle, pembrolizumab was discontinued given the availability of hysterectomy as a safe and effective alternative treatment option.

Immunotherapy is emerging as a promising cancer treatment option with the PD-1/PD-L1 pathway one of the most common therapeutic targets. Based on the two cases reported to date, anti-PD-1 therapy may achieve a complete response in patients with early stage PSTT, a tumor affecting women of childbearing age, although appropriate patient selection remains to be defined. In the case reported by Polnaszek *et al.,* pathology demonstrated a tumor proportion score of 100 % whereas in our case, PD-L1 was expressed in 80 % of tumor cells. A minimum tumor proportion score for patient selection may be considered in future pending outcomes from further cases.

Ghorani *et al.* report four patients with chemotherapy-resistant GTN treated with pembrolizumab, including two patients with PSTT (Ghorani et al., 2019). Response to pembrolizumab was observed in the patient whose tumor demonstrated 90 % PD-L1 expression, was HLA-G positive, and had tumor infiltrating lymphocytes. In contrast, no response was seen in the patient with a mixed PSTT / epithelioid trophoblastic tumor (ETT) with > 90 % PD-L1 expression but lacking tumour-infiltrating lymphocytes and HLA-G expression. Whether the absence of HLA-G and tumour-infiltrating lymphocytes predicts response to pembrolizumab remains under investigation.

In highly selected patients, pembrolizumab may be considered as an alternative to radical surgery for those desiring fertility preservation. The optimal dosing, schedule, and need for consolidation therapy remain undefined. Nevertheless, a complete response was achieved in the two cases reported to date, after one to three cycles of pembrolizumab. Ongoing studies are evaluating fertility, pregnancy outcomes, and long-term oncologic outcomes, following immunotherapy in patients with GTN.

Written informed consent was obtained from the patient for publication of this case report and accompanying images.

## CRediT authorship contribution statement

**Pierre Descargues:** Writing – review & editing, Writing – original draft, Investigation. **Lua R. Eiriksson:** Writing – review & editing. **Pascal Rousset:** Writing – review & editing, Investigation. **Adrien Msika:** Writing – review & editing. **Benoit You:** Writing – review & editing, Investigation. **Alexis Trecourt:** Writing – review & editing, Investigation. **Jerome Massardier:** Writing – review & editing, Investigation. **Touria Hajri:** Writing – review & editing, Investigation. **Bruno Borghese:** Writing – review & editing, Investigation. **François Golfier:** Writing – review & editing, Investigation. **Pierre-Adrien Bolze:** Writing – review & editing, Supervision, Investigation. **Jérôme Alexandre:** Writing – review & editing, Investigation.

## Declaration of competing interest

The authors declare that they have no known competing financial interests or personal relationships that could have appeared to influence the work reported in this paper.
